# *Francisella* sp., a Close Relative of *Francisella orientalis*, Causing Septicemia with Cholestatic Hepatitis in a Patient with Anti-Interferon-γ (IFN-γ) Autoantibodies

**DOI:** 10.3390/tropicalmed7020025

**Published:** 2022-02-11

**Authors:** Rattagan Kajeekul, Somchai Insiripong, Athita Riwlord, Suleeporn Poomchuchit, Anusak Kerdsin

**Affiliations:** 1 Department of Medicine, Maharat Nakhon Ratchasima Hospital, Nakhon Ratchasima 30000, Thailand; rattagan_k@yahoo.com (R.K.); chaikorat@gmail.com (S.I.); 2 Clinical Microbiology Laboratory, Department of Medical Technology, Maharat Nakhon Ratchasima Hospital, Nakhon Ratchasima 30000, Thailand; tatatal.2012@gmail.com; 3 Department of Community Health, Faculty of Public Health, Chalermphrakiat Sakon Nakhon Province Campus, Kasetsart University, Sakon Nakhon 47000, Thailand; suleepon.po@ku.th

**Keywords:** *Francisella philomiragia*, genome, anti-interferon-γ autoantibodies

## Abstract

*Francisella* is an intracellular, fastidious, Gram-negative bacterium that is difficult to identify using routine microbiological methods in the laboratory. We studied the isolation of *Francisella* sp. (strain IDAMR664) from the blood of a patient with anti-interferon-γ (IFN-γ) autoantibodies who presented with septicemia and cholestatic hepatitis. Analysis of the strain IDAMR664 genome sequence revealed the isolate was closely related to the strain GA01-2794 that had been isolated from a human in the USA. In addition, it was clustered with *F. orientalis*, a fish pathogen. The isolate contained several virulence factors and had *Francisella* pathogenicity island pattern no. 3.

## 1. Introduction

*Francisella* (*F*.) is a Gram-negative, pleomorphic coccobacillus. This genus comprises 11 different species [1]. Whole genome maximum likelihood phylogeny is classified *Francisella* into four clades: Clade 1 consists of *F. tularensis*, *F. novicida*, *F. hispaniensis*, *F. persica,* and *F. opportunistica;* Clade 2 comprises *F. philomiragia*, *F. noatunensis*, *F. orientalis*, and *F. salimarina*; while Clades 3 and 4 consist of *F. endociliophora* and *F. halioticida*, respectively, as representative species [1].

Among these four clades, *F. tularensis* is highly pathogenic, with the agent of tularemia being isolated from humans and many animal species, including mammals, birds, fish, amphibians, arthropods, and protozoa [2]. In addition to *F. tularensis*, there have also been reports of *F. philomiragia*, *F. hispaniensis*, *F. novicida*, *F. novicida*-like, and *F. halioticida*-like causing infection in humans, and they have been related to water-borne infections [3,4,5,6,7,8,9]. Pathogenesis of the *Francisella* species is not well-understood compared to *F. tularensis*. It has been suggested that inhalation and/or ingestion of contaminated water may increase the risk of infection in humans, especially immunocompromised patients [3,4,5,6,7,8,9].

In Thailand, *Francisella* infection has never been reported. In this study, we report a case of clinical *Francisella* sp. (a close relative of *F. orientalis*) septicemia presented in a male with intermittent fever, cholestatic hepatitis, and newly diagnosed anti-interferon-γ (IFN-γ) autoantibodies. Genomic analysis was also conducted on the isolate to explore its genome characteristics, virulence features, and relationship with other *Francisella* strains in the Genbank database.

## 2. Materials and Methods

### 2.1. Ethics

This study was reviewed and approved by the Ethics Committee of Maharat Nakhon Ratchasima Hospital, Thailand. The authors reviewed the medical record under the protocol approved by the Committees and conducted the study according to the Principles of the Declaration of Helsinki. The approval number was 127/2021.

### 2.2. Clinical Data Collection

The patient was an elderly Thai male with *Francisella* septicemia and was treated in Maharat Nakhon Ratchasima Hospital (NRH), a referral center in northeastern Thailand. His medical record was reviewed and the following variables were collected: (1) baseline characteristics (age, gender, underlying disease, history of illness, and current medication); (2) clinical presentations (signs (objective evidence), symptoms (subjective evidence), source of infection, and complications); (3) laboratory-related data (complete blood count, blood chemistry, hemoculture, bacterial isolate identification, and antibiotic susceptibility test results); and (4) treatment, length of hospital stay, and outcome.

### 2.3. Bacterial Isolation, Identification, and Antimicrobial Resistance Testing

A bacterial isolate (IDAMR664) was obtained from hemoculture of the patient. It was cultured on sheep blood agar at 37 °C for 24 h. Conventional biochemical tests were applied for the preliminary identification of this organism at the hospital [10]. Isolate no. IDAMR664 was sent to the Public Health Microbiology Laboratory Service, Faculty of Public Health, Kasetsart University, Chalermphrakiat Sakon Nakhon province campus, Thailand for further identification. 16S rRNA gene sequencing with the primer pair 27F and 1492R was performed, as described elsewhere [11]. Antimicrobial susceptibility was carried out using the broth microdilution technique to determine the minimum inhibitory concentrations (MICs) of gentamicin, tetracycline, ciprofloxacin, and chloramphenicol according to the Clinical and Laboratory Standard Institute (CLSI) guidelines [12]. Interpretation of susceptibility was based on the clinical breakpoints of *F. tularensis* following the CLSI M45 guidelines [12].

### 2.4. Whole-Genome Sequencing and Analysis

The whole genome sequencing (WGS) of this isolate was carried out on the Illumina platform at MicrobesNG (Birmingham, UK). Briefly, bacterial genomic DNA was extracted using ZymoBIOMICS DNA Kits (Zymo Research, CA, USA) and quantified in triplicate using the Quantity dsDNA HS assay in an Eppendorff AF2200 plate reader (Eppendorff, Stevenage, UK). Genomic DNA libraries were prepared using a Nextera XT Library Prep Kit (Illumina, San Diego, CA, USA) following the manufacturer’s protocol. Pooled libraries were quantified using a Kapa Biosystems Library Quantification Kit for Illumina on a Roche light cycler 96 qPCR machine. Libraries were sequenced on an Illumina instrument using a 250 bp paired-end protocol.

Illumina short reads were adapter-trimmed using Trimmomatic 0.30 with a sliding window quality cutoff of Q15 [13]. Then, de novo assemblies were performed using SPAdes version 3.7 and contigs were annotated using Prokka 1.11 [14,15]. Species determination was confirmed through average nucleotide identity (ANI) [16] and KmerFinder-3.2 [17] using the whole genome sequence. Clade-2 *Francisella* representative species (*F. philomiragia* ATCC25015, *F. orientalis* FSC771, *F. noatunenesis* FSC846, *F. salimarina* CHUGA-F75, and *Francisella* sp. GA01-2794) were used as reference genomes for the ANI comparisons [1,18]. Antimicrobial resistance genes were detected using ResFinder 4.1 and the Comprehensive Antibiotic Resistance Database (CARD 2.0) [19,20]. Analysis of virulence factors was conducted using the VFDB database [21]. The IDAMR644 genome was analyzed using a *Francisella* pathogenicity island (FPI) with a specific FPI gene content, including oligopeptide transport (OppABCDF), spermidine/putrescine biosynthesis (SpeADE, AguAB), unique lipopolysaccharide synthesis (FTT0794-FTT0796, FTT1188, FTT1453c, FTT1454c, FTT1458c), and four RM system types.

In total, 25 representative clade-2 *Francisella* genomes deposited in the Genbank database were retrieved and used to analyze our isolate genome. A reference genome-based single nucleotide polymorphism (SNP) strategy with CSI phylogeny was conducted [22]. The phylogenetic tree was visualized using iTOL V4 [23]. *F. philomiragia* ATCC25015, a type of strain (accession no. CP010019), was used as the reference sequence for SNP analysis.

### 2.5. Accession Number

The genome sequence of the *Francisella* sp. strain IDAMR644 was deposited in the NCBI GenBank under the Bioproject accession number PRJNA758025, with an accession number of JAINFB000000000.

## 3. Results

In 2018, a 62-year-old male farmer was diagnosed with tuberculous lymphadenitis in the left inguinal area. The diagnosis was based on the histopathology of the lymph node tissue that showed suppurative granulomatous and positive AFB staining. Unfortunately, the lymph node tissue was not sent for mycobacterial culture. He received oral isoniazid (INH 300 mg), rifampicin (RFP 600 mg), pyrazinamide (PZA 1500 mg), and ethambutol (ETB 1000 mg) daily for 2 months (induction phase) and then continued only with INH and RFP for 4 months (maintenance phase). After completing anti-tuberculosis drugs for 6 months, the lymph nodes in the left inguinal area subsided.

In January, 2021, he went to a community hospital with acute high-grade fever, shivering, and jaundice. He was diagnosed with *Salmonella* group D septicemia and received 2 gm/day intravenous ceftriaxone for 14 days. On the third day of ceftriaxone therapy, he defervesced; meanwhile, other symptoms were unremarkable. He returned to the same hospital 2 weeks later because of intermittent fever, progressive jaundice, and significant weight loss of 11 kg in 4 months. He denied any recent travel outside the province and had had no contact with wild animals nor sick individuals, but he was constantly exposed to stagnant water because he was a farmer.

The physical examination revealed only fever and jaundice, with everything else unremarkable. The differential diagnoses for these presentations might include disseminated mycobacterial infection (recurrent), melioidosis, leptospirosis, rickettsiosis, and hepatobiliary tract infection. The blood test indicated a complete blood count of 7.3 g/dL hemoglobin, 23.1% hematocrit, 14.1 × 10^9^/L white blood cell (WBC) count (75.2% neutrophils, 19.0% lymphocytes, 4.6% monocytes, 0.4% eosinophils, and 0.8% basophils), and a platelet count of 153 × 10^9^/L. The liver function test revealed 70 U/L aspartate aminotransferase (AST), 43 U/L alanine transaminase (ALT), 390 U/L alkaline phosphatase (ALP), 5.4 mg/dL total bilirubin, 3.5 mg/dL direct bilirubin, 2.4 g/dL albumin, and 5.2 g/dL globulin, whereas the blood sugar was 110 mg/dL. The blood tests for anti-HIV, *Orientia tsutsugamushi* antibodies, and for melioidosis antibody titer were negative. Leptospira IgG and IgM antibodies measured using immunochromatography were negative.

The patient was referred to the provincial Maharat NRH, a tertiary referral hospital, for further investigation and treatment. He received empirical therapy with intravenous ceftriaxone at 2 g/day. The chest and abdomen computerized tomography that can be used to identify the source of infection showed no significant abnormalities. Anti-IFN-γ antibodies were tested using the enzyme-linked immunosorbent assay (ELISA) method because he had had sequential infections consisting of mycobacterial infection and salmonellosis, indicating the suspicion of an impaired cell-mediated immune response. The result was positive.

The blood culture was repeated for two bottles, yielding Gram-negative coccobacilli. Pin-point colonies appeared on sheep blood and chocolate agar plates within 24 h. Based on conventional biochemical tests, a *Haemophilus*-like organism was suspected. However, from 16S rRNA gene sequencing, the isolate was identified as *F. philomiragia/F. orientalis*. Antimicrobial susceptibility revealed that this isolate was susceptible to ciprofloxacin (MIC = 0.016 mg/mL), gentamicin (MIC = 0.25 mg/mL), chloramphenicol (MIC = 2 mg/mL), and tetracycline (MIC = 0.5 mg/mL).

**Figure 1:**
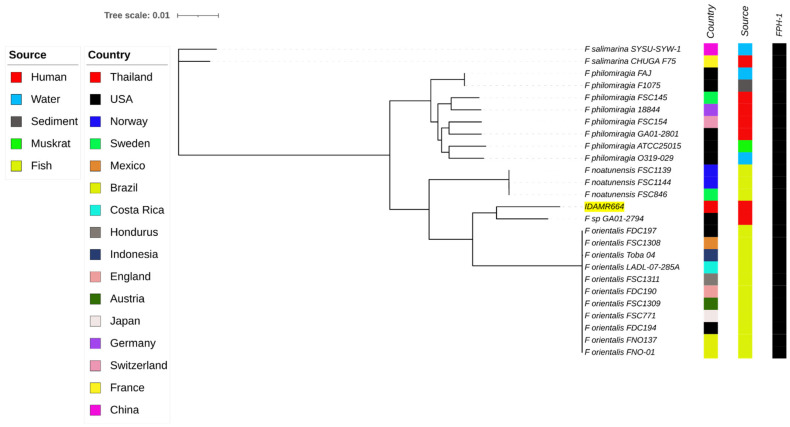
Whole-genome phylogeny analysis of *Francisella* clade-2 species generated using CSI phylogeny and visualized with an interactive life tool tree. *Francisella* strain IDAMR664 in this study is highlighted in yellow. Presentation of the antimicrobial-resistance gene, FPH-1, is shown by filled squares.

Genomic analysis of the IDAMR664 using KmerFinder suggested the isolate was *F. orientalis*. The ANI values between the strain IDAMR664 and *F. philomiragia* ATCC25015, *F. noatunenesis* FSC846, *F. salimarina* CHUGA-F75, *F. orientalis* FSC771, and *Francisella* sp. GA01-2794 were 93.12%, 94.02%, 88.96%, 95.37%, and 96.81%, respectively, that were all above the species cut-off level of 95%. Our isolate *Francisella* sp. IDAMR644, *Francisella* sp. GA01-2794, and *F. orientalis* may be considered as a single species, which correlates with ANI cut-off values of 95% according to a previous description [1]. However, the genomic comparison revealed that our isolate was more closely related to the *Francisella* sp. strain GA01-2794, which was isolated from a human in the USA, than to the *F. orientalis* cluster (Figure 1). In addition, both isolates were related to *F. orientalis* (Figure 1). Alignment of genomes between IDAMR664 and GA01-2794 using the Mauve software (version 20150226) identified 168 coding sequences (CDS) of IDMAR664 that differed from GA01-2794 (Table S1 and Supplementary File S1). Among the variable regions detected in IDAMR664, nine large regions were identified in this isolate compared with GA01-2794. BLASTN analysis of these nine regions demonstrated that Region 1 (86,696 bp) was unique to our isolate, not being found in any *Francisella* species, nor in other organisms. This region contained 80 genes or coding sequences, such as a bacteriophage structure and enzymes, hypothetical proteins, and restriction–modification enzymes (Table S1).

Acquired antimicrobial resistance genes were not found in the genome of our isolate, IDAMR664, based on ResFinder 4.1. However, CARD analysis revealed the FPH-1 gene, a carbapenem-hydrolyzing class A-β-lactamase (identity 93.99%.) All *Francisella* clade-2 genomes analyzed in this study also contained the FPH-1 gene (Figure 1). The FPH-1 encoding the carbapenemase could lead to resistance to beta-lactam antibiotics.

The VFDB analysis indicated there were many virulence factors in the genome of the IDAMR664 isolate (Table S2). The isolate contained type IV pili, FsaP, and porcine attaching-effacing associated protein for adherence and invasion, Types 4 and 6 secretion systems (T4SS, T6SS), iron uptake systems (*Francisella* siderophore, pyoverdin, and ferrous iron-transport system), intracellular survival associated factors (acid phosphatase, DipA, OmpA, and RipA), and other factors, such as capsule α-hemolysin, urease, and enolase. Analysis of the FPI pattern with a specific gene set revealed that IDAMR664 had FPI pattern no. 3. It presented as OppABCDF and a type III RM system [24].

On the fifth day of ceftriaxone therapy, the fever disappeared, so ceftriaxone was continued until 14 days. Then, ceftriaxone was switched to 1000 mg/day oral ciprofloxacin for 2 weeks, based on antimicrobial drug susceptibility testing. After completion of the antimicrobial chemotherapy, the patient did not have a recurrence of fever and the result of the liver function test was normal.

A literature review of English publications revealed that 21 cases of clade-2 *Francisella* species infection had been reported [25,26,27,28,29,30,31]. As shown in Table 1, the age of the previous patients varied, but half of them were young and they often had underlying morbidities. The infection risk factors are chronic granulomatous disease in young patients, while older patients presented with hematogenous malignancy, organ transplant and receiving immunosuppressive drugs, anti-interferon-γ (IFN-γ) autoantibodies, and saltwater near-drowning. The clinical presentations varied, including pneumonia, bacteremia, meningitis, adenitis, and peritonitis.

## 4. Discussion

*Francisella* species other than *F. tularensis*, are rare, opportunistic pathogen in humans, mainly infecting immunocompromised hosts and related to water exposure [3,4,5,6,7,8,9]. Our case is a first report of *Francisella* sp. bacteremia with cholestatic hepatitis, consistent with disseminated infection, but unfortunately, a liver biopsy was not performed. In this case, the male patient had not lived near the seacoast or been exposed to seawater, brackish water, or salt-water, which have been detailed as exposure sources of *F. philomiragia*, *F. hispaniensis*, *F. novicida*, *F. novicida*-like, and *F. halioticida*-like [32]; however, the patient’s history of repeated exposure to stagnant water may have been the route of infection. In addition, our isolate was closely related to the strain GA01-2794 that also was related to strains of *F. orientalis* and may be considered as *F. orientalis* based on its ANI cut-off value; however, further confirmation is needed. *F. orientalis* is a fish pathogen indicated that its habitat is an aquatic environment [33]. Collectively, the information on the patient could suggest that an aquatic source may have been the reservoir of our isolate; however, further investigation is necessary. In this case, the risk factor was anti-interferon-γ (IFN-γ) autoantibodies. Anti-IFN-γ autoantibodies, known as adult-onset immunodeficiency, lead to opportunistic infections, mainly by intracellular pathogens [34,35,36]. IFN-γ is secreted by activated T cells, natural killer (NK) cells, and macrophages. It can bind to the specific receptor and activate the Janus-activated kinase (JAK)-STAT pathway. This JAK-STAT signal harmonizes the transcriptional activation of several genes and mediates various biological responses against intracellular pathogens [37]. A high titer of anti-IFN-γ autoantibodies that can block the binding of IFN-γ to its receptor has been associated with various opportunistic infections. Likewise, anti- IFN-γ autoantibodies were supposed to account for *Francisella* infection in our case.

To date, there is no standard therapy for *Francisella* infection; β-lactam should be avoided in *Francisella* infection because it is well-known that it can produce Class A carbapenemase (FPH-1 or FTU-1) [38,39,40]. This enzyme hydrolyzes penicillins and the narrow-spectrum cephalosporins, such as cephalothin [39]. All *Francisella* clade-2 samples analyzed in the current study harbored class A FPH-1 carbapenemase and this enzyme had sequence homology to FTU-1 β-lactamase from *F. tularensis* [39,40]. FPH-1 confers a much broader spectrum and higher levels of resistance to various β-lactam antibiotics than FTU-1 β-lactamase that include penicillins, ampicillin, oxacillin, amoxicillin, piperacillin, ticarcillin, cephalosporins, monobactam, aztreonam, and carbapenem [39,40]. Surprisingly, our patient responded well to ceftriaxone, although there was no information concerning its sensitivity. The CLSI guidelines do not recommend testing the sensitivity of *Francisella* to β-lactam. The pathogenicity and virulence features of *Francisella* species depend on the FPI. Four major patterns of the FPI gene content have been identified in *Francisella* genomes [24]. As mentioned in another report, IDAMR664 shows FPI pattern no. 3 that is similar to those of other *F. philomiragia* strains, with the presence of OppABCDF and either type I or III RM systems [24]. Other virulence factors, such as type IV pili—involved in bacterial adhesion to host cells and biofilm formation as well as FPI-related genes, such as oligopeptide ABC transporter (OppABCDF) or T6SS (*igl, pdp*)—may have a role in the signal transduction and pathogenesis that were found in our isolate IDAMR664 [41]. The presence of other virulence-related genes (*feoB*, *fupA*, *fslE*, *T4SS*, *fsaP*, *hylB*, *paa*, and *ureABC*) in this isolate genome suggests that these functions are essential for survival in either the host or environment.

## 5. Conclusions

This was the first reported case involving an elderly Thai male admitted due to *Francisella* sp. (a close relative of *F. orientalis*) septicemia with cholestatic hepatitis and a history of mycobacterial infection and salmonellosis that often occur in patients with cell-mediated defects. Most of these infections are usually found in HIV-positive people. Nevertheless, this patient was anti-HIV negative, so other acquired immunodeficiency states with T-cell defects, such as autoantibody to interferon-γ, should raise awareness and heighten suspicion that has been rarely reported.

Analysis of the strain IDAMR664 genome sequence revealed the isolate was closely related to the strain GA01-2794 that has been isolated from a human in the USA. The isolate contained several virulence factors and it had *Francisella* pathogenicity island pattern no. 3.

## Figures and Tables

**Table 1:** Review of 22 clade-2 *Francisella* species reported cases in the literature.

Pathogen	Reference	Location	Age (yr)	Gender	Risk Factors	Clinical Presentation	Source of Isolate	Treatment	Outcome
*F. philomiragia*	Wenger et al., 1989 [25]	California, USA	18	Male	CGD	Pneumonia	Lung biopsy	Chloramphenicol, sulfisoxazole, penicillin G	Improvement
		New York, USA	NA	Male	Near-drowning	NA	Blood	NA	NA
		California, USA	39	Male	Near-drowning	Pneumonia	Blood	Cephalothin, gentamicin	Death
		Switzerland	6	Male	CGD	Fever	Blood, bone marrow, ascites fluid	Cefotaxime, amikacin, trimethoprim-sulfamethoxazole	Death
		Pennsylvania, USA	68	Female	Agnogenic myeloid metaplasia	Fever	Blood	Cephalexin, tobramycin	Improvement
		Connecticut, USA	86	Female	Near-drowning	Pneumonia	Blood	Oxacillin, gentamicin	Improvement
		Connecticut, USA	75	Male	Near-drowning	Pneumonia	Blood	Cephalothin, gentamicin	Improvement
		New York, USA	5	Male	CGD	Fever	Blood	NA	NA
		California, USA	12	Female	CGD	Pneumonia	Lung biopsy	Erythromycin, rifampin	NA
		New Mexico, USA	34	Female	NA	Peritonitis	Ascites fluid	Clindamycin, gentamicin	Improvement
		Virginia, USA	28	Male	Near-drowning	Sepsis	Blood	NA	NA
		New York, USA	47	Female	Hodgkin’s disease	Sepsis	Blood, pericardial fluid	Erythromycin, tobramycin, trimethoprim-sulfamethoxazole	Improvement
		Massachusetts, USA	16	Male	CGD	Meningitis	Cerebrospinal fluid	Vancomycin, gentamicin, others	Improvement
	Sicherer et al., 1997 [26]	Maryland, USA	19	Male	CGD	Fever	Blood	Cefotaxime, gentamicin, ciprofloxacin	Improvement
	Mailman et al., 2005 [27]	Nova Scotia, Canada	10	Male	CGD	Sepsis, adenitis, pulmonary nodule	Lymph node	Ciprofloxacin	Improvement
	Friss-Moller et al., 2004 [28]	Turkey	25	Male	CGD	Pneumonia	Blood	Cefuroxime, ciprofloxacin, meropenem, others	Death
	Relich et al., 2015 [3]	Indiana, USA	63	Female	Renal transplant	Pneumonia	Blood	Piperacillin-tazobactam, doxycycline	Improvement
	Kreitmann et al., 2015 [29]	France	58	Male	Myeloproliferative disorder, Sweet’ s syndrome	Fever	Blood	Cefotaxime, gentamicin, ciprofloxacin	Improvement
	Robles-Marhuenda et al., 2018 [30]	Spain	20	Male	CGD	Pneumonia	Blood	Amoxicillin, clarithromycin, levofloxacin	Improvement
*F. salimarina*	Hennebique et al., 2022 [31]	France	76	Male	acute myelomonocytic leukemia	Fever with skin ulcer	Blood	Doxycycline, sulfamethoxazole/trimethoprim	Improvement
*Francisella* sp. GA01-2794	Wenger et al., 1989 [25]	Colorado, USA	39	Male	NA	Fever	Pleural fluid	None	Improvement
*Francisella* sp. IDAMR664	Present case	Thailand	62	Male	anti-interferon-γ autoantibodies	Fever with jaundice	Blood	Ceftriaxone, ciprofloxacin	Improvement

NA = Not Applicable; CGD = chronic granulomatous disease.

## Data Availability

No new data were created or analyzed in this study. Data sharing is not applicable to this article.

## Supplementary Materials

The following are available online at https://www.mdpi.com/article/10.3390/tropicalmed7020025/s1, Table S1: Region of difference in *F. philomiragia* stran IDAMR664, Table S2: Predicted virulence factors based on the VFDB analysis in *F. philomiragia* strain IDAMR664. Supplementary File S1: Genomic comparison of *F. philomiragia* strains IDAMR664 and GA01-2794 using the Mauve software.
