# Supplementary material for: Francisella sp., a Close Relative of Francisella orientalis, Causing Septicemia with Cholestatic Hepatitis in a Patient with Anti-Interferon-γ (IFN-γ) Autoantibodies
Source: Trop Med Infect Dis. 2022 Feb 11;7(2):25. doi: 10.3390/tropicalmed7020025 (PMC8874608; doi:10.3390/tropicalmed7020025)
Supplement: Supplementary file 1 [file tropicalmed-07-00025-s001.zip › Supplemental file 1.pdf]

Supplemental file 1: Genomic comparison of *Francisella philomiragia* strains IDAMR664, ATCC25015 and GA01-2794 using the Mauve software and CGview server tool

Genomic comparison among IDAMR664, GA01-2794, and ATCC25015 using the Mauve software [1] identified 168 coding sequences (CDS) of IDMAR664 that differed from GA01-2794 and ATCC25015 (Figure S1 and Table S1). As depicted in Figure S1, the contigs boundaries of each genome analyzed are represented using red lines and the conserved, distinct, locally collinear blocks (LCBs) for all strains are different colors with positional agreement between scaffolds tiled against the respective closed chromosomal sequences. Conserved regions were mostly found in these 3 genomes analyzed using CGview [2]; however, variable regions were observed in these genomes (Figure S2). The variable sequences (CDS) detected in our isolate genome are summarized in Table S1.

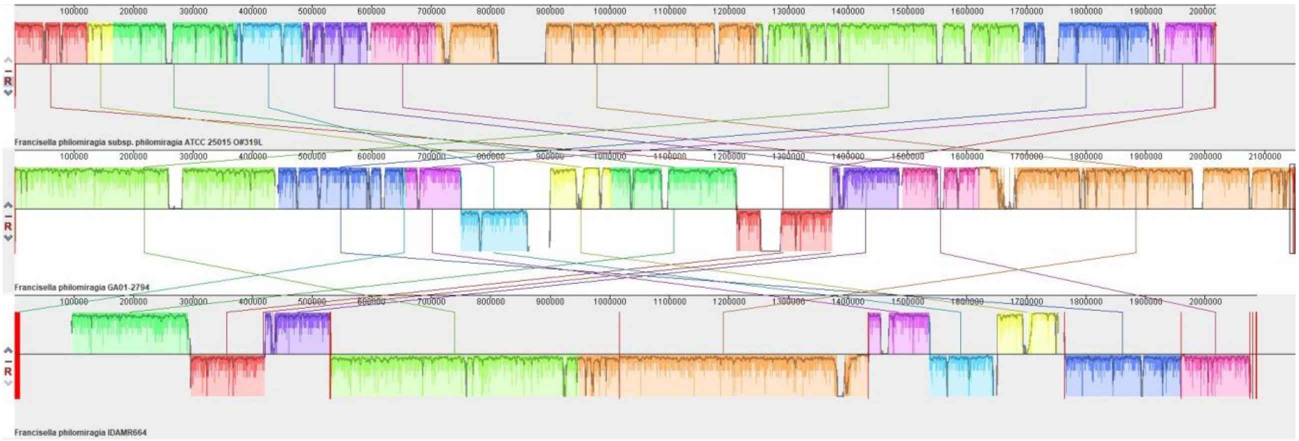

Figure S1. Multigenome comparisons among *F. philomiragia* strains ATCC25015, GA01-2794, and IDAMR664 performed using the Mauve software. Each color region refers to a locally collinear block (LCB). Colors are arbitrarily assigned to each LCB by the software. The vertical peaks in each LCB denote the variance of conservation. The LCBs below the centerline of the genomes are in reverse complement orientation. The reference genomes, ATCC25015, were compared with GA01-2794 and IDAMR664.

Among the variable regions detected in IDAMR664, 9 large regions were identified in this isolate compared with GA01-2794 and the type stain ATCC25015. BLASTN analysis of these 9 regions demonstrated that region 1 (86,696 bp) was unique to our isolate, not being found in any *Francisella* species, nor in other organisms. This region contained 80 genes or coding sequences, such as bacteriophage structure and enzymes, hypothetical proteins, restriction-modification enzymes (Table S2). Region 2 (4,531 bp) was detected in *F. opportunistica*, *F. noatunensis* subsp. *orientalis*, and *F. marina* with sequence coverages of 70%, 67%, and 62%, respectively, but was not found in *F. philomiragia*. Region 3 (5,426 bp) was mostly present in *F. salimarina* CHUGA-F75 (87% coverage). Region 4 (5,867 bp) was detected in *F. noatunensis* subsp. *orientalis* with 100% coverage, whereas it was mostly present in *F. salimarina* and *F. marina* with 90% coverage for both, and 80% coverage in *F. philomiragia* strains O#319-067, O#319-029, and ATCC25017. Region 5 (18,967 bp) was mostly

present in *F. philomiragia* strain FSC153 (93% coverage) and *F. noatunensis* subsp. *orientalis* (85% sequence coverage). Region 6 (13,508 bp) was partially present in *F. opportunistica*, *F. tularensis* subsp. *novicida*, and *F. uliginis* with 57%, 43%, and 42% coverages, respectively. Region 7 (6,550 bp) was found in *F. philomiragia* (GA01-2801) and *F. tularensis* subsp. *novicida* (U112) with 79% coverage for both. Region 8 (11,130 bp) was partially detected in *F. marina* (42% coverage), whereas region 9 (10,609 bp) was mostly present in *F. tularensis* subsp. *novicida* (TCH2015) with 79% coverage.

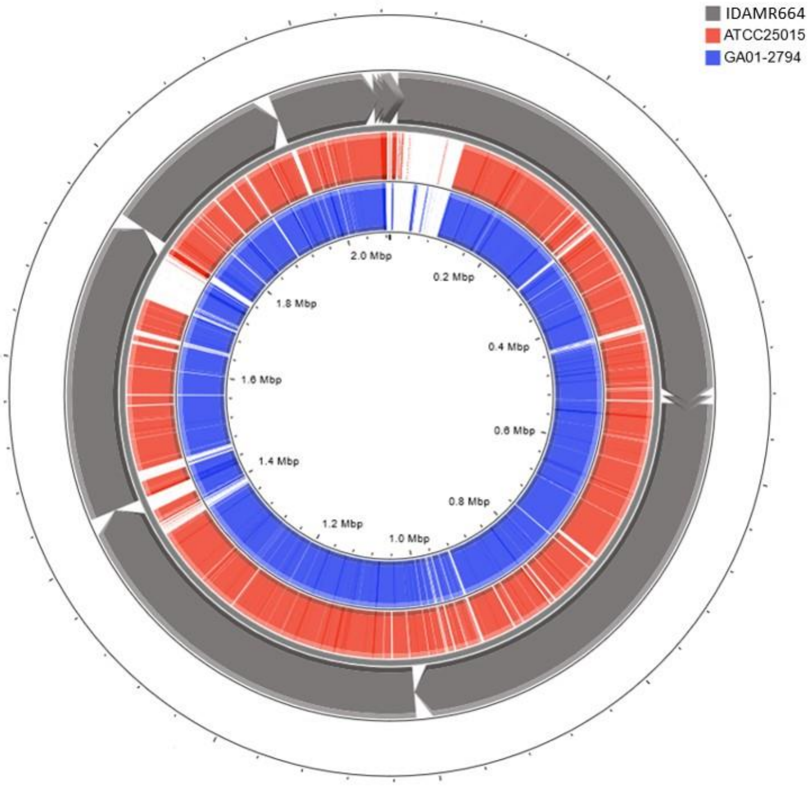

Figure S2. Comparative genomic circular map of *F. philomiragia* strain IDAMR664 constructed using BRIG v0.95. The features are (ring from center to outside): ring 1 is genome size; rings 2–4 are comparative genomic maps of *F. philomiragia* strains GA01-2794, ATCC25015, and IDAMR664, respectively, with the ATCC25015 genome as the reference. Blank spaces in the ring represent matches with less than 50% identity to the reference genome (ATCC25015).

References

1. Darling, A.C.; Mau, B.; Blattner, F.R.; Perna, N.T. Mauve: multiple alignment of conserved genomic sequence with rearrangements. *Genome Res.* **2004**, *14*, 1394-1403.
2. Stothard, P.; Wishart, D.S. Circular genome visualization and exploration using CGView. *Bioinformatics* **2005**, *21*, 537-539.
