# Supplementary figures and images for: Francisella sp., a Close Relative of Francisella orientalis, Causing Septicemia with Cholestatic Hepatitis in a Patient with Anti-Interferon-γ (IFN-γ) Autoantibodies
Source: Trop Med Infect Dis. 2022 Feb 11;7(2):25. doi: 10.3390/tropicalmed7020025 (PMC8874608; doi:10.3390/tropicalmed7020025)

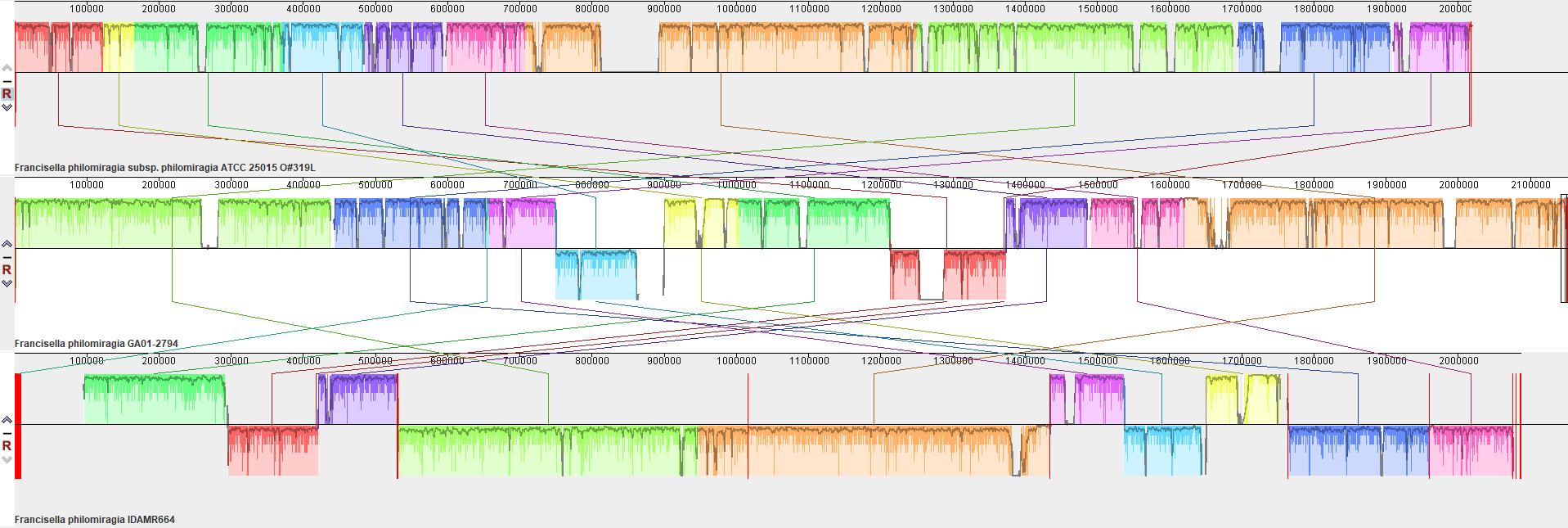

Supplement: Supplementary file 1 [file tropicalmed-07-00025-s001.zip › Figure S1_Mauve comparison.jpg]

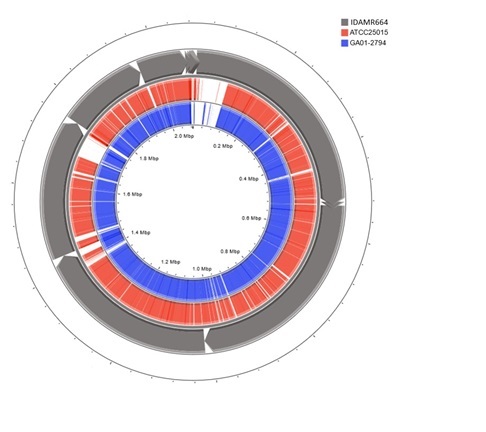

Supplement: Supplementary file 1 [file tropicalmed-07-00025-s001.zip › Figure S2_CG genome.jpg]
